# The “extreme phenotype approach” applied to male breast cancer allows the identification of rare variants of ATR as potential breast cancer susceptibility alleles

**DOI:** 10.18632/oncotarget.28358

**Published:** 2023-02-07

**Authors:** Martin Chevarin, Diana Alcantara, Juliette Albuisson, Marie-Agnès Collonge-Rame, Céline Populaire, Zohair Selmani, Amandine Baurand, Caroline Sawka, Geoffrey Bertolone, Patrick Callier, Yannis Duffourd, Philippe Jonveaux, Yves-Jean Bignon, Isabelle Coupier, François Cornelis, Christophe Cordier, Monique Mozelle-Nivoix, Jean-Baptiste Rivière, Paul Kuentz, Christel Thauvin, Romain Boidot, François Ghiringhelli, Marc O'Driscoll, Laurence Faivre, Sophie Nambot

**Affiliations:** ^1^Inserm UMR 1231 GAD Génétique des Anomalies du Développement, Université de Bourgogne, Dijon, France; ^2^Unité Fonctionnelle Innovation diagnostique dans les maladies rares, laboratoire de génétique chromosomique et moléculaire, Plateau Technique de Biologie, CHU Dijon Bourgogne, Dijon, France; ^3^Human DNA Damage Response Disorders Group, University of Sussex, Genome Damage and Stability Centre, Brighton, United Kingdom; ^4^Service d’Oncogénétique, Centre Georges François Leclerc, Dijon, France; ^5^Département de biologie et pathologie des tumeurs, Centre Georges François Leclerc, Dijon, France; ^6^Oncobiologie Génétique Bioinformatique, PCBio, CHU Besançon, Besançon, France; ^7^Centre de Génétique et Centre de Référence Maladies Rares Anomalies du Développement de l’Interrégion Est, Hôpital d’Enfants, CHU Dijon Bourgogne, Dijon, France; ^8^Fédération Hospitalo-Universitaire Médecine Translationnelle et Anomalies du Développement (FHU TRANSLAD), CHU Dijon Bourgogne et Université de Bourgogne-Franche Comté, Dijon, France; ^9^Laboratoire de Génétique Médicale, INSERM U954, Hôpitaux de Brabois, Vandoeuvre les Nancy, France; ^10^Laboratoire d’Oncologie Moléculaire, Centre Jean Perrin, Clermont-Ferrand, France; ^11^Unité d’Oncogénétique, ICM Val d’Aurel, Montpellier, France; ^12^Université Bordeaux, IMB, UMR 5251, Talence, France; ^13^Service d’imagerie diagnostique et interventionnelle de l’adulte, Hôpital Pellegrin, CHU de Bordeaux, France; ^14^UF6948 oncogénétique, CHRU de Strasbourg, France; ^15^Service de génétique, CHU-Reims, France; ^16^Département d’oncologie médicale, INSERM LNC U1231, Centre Georges François Leclerc, Dijon, France

**Keywords:** male breast cancer, genetic predisposition to cancer, exome sequencing, *ATR*, extreme phenotype

## Abstract

In oncogenetics, some patients could be considered as “extreme phenotypes”, such as those with very early onset presentation or multiple primary malignancies, unusually high numbers of cancers of the same spectrum or rare cancer types in the same parental branch. For these cases, a genetic predisposition is very likely, but classical candidate gene panel analyses often and frustratingly remains negative. In the framework of the EX^2^TRICAN project, exploring unresolved extreme cancer phenotypes, we applied exome sequencing on rare familial cases with male breast cancer, identifying a novel pathogenic variant of *ATR* (p.Leu1808*). *ATR* has already been suspected as being a predisposing gene to breast cancer in women. We next identified 3 additional *ATR* variants in a cohort of both male and female with early onset and familial breast cancers (c.7762-2A>C; c.2078+1G>A; c.1A>G). Further molecular and cellular investigations showed impacts on transcripts for variants affecting splicing sites and reduction of ATR expression and phosphorylation of the ATR substrate CHEK1. This work further demonstrates the interest of an extended genetic analysis such as exome sequencing to identify very rare variants that can play a role in cancer predisposition in extreme phenotype cancer cases unexplained by classical cancer gene panels testing.

## INTRODUCTION

In many patients with a personal or familial history strongly suggestive of a Mendelian predisposition to cancer, screening of known cancer predisposition genes often remains negative, compelling patients and care providers into an unfavorable situation regarding genetic counselling and family screening. In the field of oncogenetics, some cases can be qualified as “extreme phenotypes”. These include patients with very early onset presentation, patients belonging to families with unusually high numbers of cancer cases of the same spectrum in the same parental branch, patients with multiple primary malignancies of the same spectrum or with early-onset in some of them or families with multiple cases of rare cancer.

During the last decade, next generation sequencing (NGS), and particularly exome sequencing (ES), has revealed the great variability of human genome [1, 2], and revolutionized the diagnosis of rare Mendelian disorders [3]. In oncogenetics, ES has allowed the identification of new susceptibility genes and the extension of the spectrum of known cancer predisposition genes [4–9]. It is now suspected that a fraction of Mendelian predisposition to cancer could result from very rare germline variants affecting yet unknown cancer predisposing genes or be due to variants of known susceptibility genes that are not included in commonly used cancer predisposition gene panels. This hypothesis has been tested in cohorts of breast and ovarian cancers, with some promising results suggested from large gene panels or ES in high-risk families of breast cancer (BC), but negative for well-established BC predisposition genes [7, 9–11]. In a translational research project named EX^2^TRICAN, we seek to extend investigations through ES in extreme phenotype cancer. Among patients included in this study, there were 3 patients from families with multiple cases of male breast cancer (MBC).

MBC accounts for less than 1% of all BC cases worldwide and among male cancers [12]. 20% of MBC cases are diagnosed in families with evidence of female breast and ovarian hereditary syndromes, additionally 20% of men with BC developed an asynchronous secondary malignancy [13]. This is higher than the estimated proportion of female breast cancer (FBC) of genetic origin and point to a more important implication of the genetic component in MBC susceptibility. Thereby, the occurrence of a MBC in a family is an indication for prescribing genetic susceptibility tests for BC, even in the absence of family history of cancer [14]. As for FBC, the two main high penetrance predisposition genes are *BRCA1* and *BRCA2*. Germline variants of *BRCA2* are responsible for about 10% of all MBC cases and around 17% of MBC cases in families with high risk of BC [15]. Germline variants of *BRCA1* are rare in unselected MBC cases (< 2%) [15–19], but the frequency increases to about 7% in MBC cases related to high risk BC families [16, 20, 21]. Pathogenic variants of the *PALB2* and *CHEK2* genes have also been identified in MBC cases [22–24]. A more recent study in the Italian population is in favor of a central role of *PALB2* in MBC susceptibility, but show low impact of *CHEK2* [25]. It has been shown that Klinefelter syndrome (MIM #400045), a sex chromosome disorder resulting of one or more extra copy of chromosome X in a male, increases the risk of MBC by 20-fold as compared to the general population [26, 27] and some cases of MBC have been reported among patients with cancer predisposition syndromes such as Li-Fraumeni (MIM #151623) (*TP53*)[16], Cowden (MIM #158350) (*PTEN*) [28, 29] and Lynch syndromes (MIM #609310, MIM #120435, MIM #614350, MIM #614337) (*MLH1, MSH2, MSH6, PMS2*) [30–33].

In one of the 3 patients with MBC included in the EX^2^TRICAN project, we identified a non-sens variant in the *ATR* gene.

*ATR* (Ataxia Telangiectasia and RAD3-related) encodes a 2644 amino acids protein belonging to the phosphatidylinositol 3-kinase-related protein kinase (PIKK) family. In coordination with ATM (Ataxia Telangiectasia-Mutated), ATR acts as an apical kinase of the DNA damage response (DRR), a complex signal transduction network that controls the integrated activation of the cell cycle checkpoint response, DNA replication fork stabilization and DNA repair pathway function [34–39]. ATM is specifically activated by DNA double strand breaks (DSBs), whilst RPA-coated single stranded DNA (or single stranded breaks; SSBs), typically generated upon DNA replication fork stalling and collapse, acts as the specifying activating signal for ATR. Furthermore, ATR plays an important role in the stability of DNA common fragile sites [40] and is involved in the regulation of centrosomes duplication [41]. Under conditions of impaired ATR function, regions of incomplete DNA replication and elevated levels of spontaneous DNA replication fork collapse generate significant levels of DNA breakage (incl. SSBs, DSBs, nicks, gaps and complex rearrangements), in a process collectively referred to as “Replication Stress (RS)”. Uncontrolled elevated RS can directly cause deletion of genetic information, chromatin exchanges and translocations. The resulting genetic instability can initiate and drive malignant transformation [40–42]. Moreover, loss of one allele of *Atr* has been shown to increases tumor incidence in mice [43].

It has been established that ATR is an essential protein for the viability of normal cells [35, 43, 44]. However, ATR functions appear to be even more critical for survival of cancer cells with activated oncogenes such as RAS, MYC and Cyclin E which themselves disrupt the normal cell cycle regulation generating high level of RS [45, 46]. Persistently high RS represents a vulnerability to unrepaired DNA damage [46]. Several studies have shown that inhibition of the ATR pathway is selectively toxic to cancer cells with high oncogene-driven replication damage [47–50] and inhibition of the functional kinase activity of ATR sensitizes cancer cells to conventional DNA damaging chemotherapies, ionizing radiation and immunotherapy [51–55]. Therefore, inhibition of the ATR pathway has been considered as a therapeutic strategy and as of year 2021, several ATR inhibitors were under testing in 39 different phase I or II clinical trials, most often in combination with other chemotherapy, targeted inhibitors or immunotherapies (https://clinicaltrials.gov/). Interestingly, one of these inhibitors was observed to induce marked and durable response rates in a subset of relapsed small cell neuroendocrine cancer patients, suggesting a potential new therapeutic line for these patients with obscure prognosis [56].

2.9% of patients included in BC studies referred in cBioPortal (https://www.cbioportal.org/) [57, 58] are carriers of somatic variants of *ATR*. For comparison, *ATM* is somatically altered in 5.2% of patients of the same studies. If considering only pathogenic or likely pathogenic variants, somatic variants rates are 0.7% and 2.4% for *ATR* and *ATM*, respectively. Of note, 8% of these somatic alterations of *ATR* are gene amplifications (Supplementary Figure 1). These lower somatic variants burden and presence of gene amplifications could also reflect the essential role of ATR in cancer cells.

Constitutional homozygous or compound-heterozygous hypomorphic variants of *ATR* cause Seckel syndrome (MIM #210600), characterized by intrauterine growth retardation and primordial dwarfism, with marked microcephaly. Seckel syndrome patient cell lines show defects in phosphorylation of ATR substrates, instability of replication forks and disruption of activation of the cell cycle G2/M checkpoint [59–61]. *ATR* expression is never null in Seckel patients, consistent with ATR’s essential role for cells viability.

Basic functions of ATR classify it in the category of tumor suppressor genes but further studies show that its role is more complex in the context of cancer. Here, using a combination of ES, direct sequencing of *ATR* in a replication cohort and prospective screening, followed by functional investigations we report the identification of new candidate variants of *ATR* as predisposing to BC, including MBC.

## RESULTS

### Identification of *ATR* variants

The first variant of *ATR* identified in an MBC case (PED2361.1) using ES in the framework of the EX^2^TRICAN project was a non-sense variant affecting exon 32 (NM_001184.3:c.5423T>G - p.Leu1808*). This variant is absent from the gnomAD database (gnomAD v2.1.1 non cancer) and the observed/expected score for loss of function of *ATR* is 0.31 (90% CI: 0.24–0.4). In fact, ATR has a negative residual-variation intolerance score of −1.64 (percentile of 2.79% ) (Genic intolerance, http://genic-intolerance.org/) [62]. The patient presented BC at age 44, and was investigated because of an initially reported family history of BC in his father. Segregation showed that the variant was inherited from his unaffected mother. Given this result, retrospective analysis of the medical file in fact confirmed no indication of BC in his father (Supplementary Figure 2).

Targeted NGS of *ATR* in the replication cohort identified a second likely pathogenic variant in another MBC case (PED3315.1), who presented BC at age 70. It was a near splice variant affecting the consensus splicing acceptor site before the last exon (NM_001184.3:c.7762-2A>C). Only one variant was reported at this position in over 236422 non-cancer alleles in the gnomAD database, it was predicted as “most probably affecting splicing” by the Human Splicing Finder prediction tool (HSF, http://umd.be/Redirect.html) and has a probability of altering splicing of 98.41% according to Splicing Prediction Pipeline (SPiP, https://sourceforge.net/projects/splicing-prediction-pipeline/).

Six additional variants of unknown significance (VUS) were identified in the replication cohort: 5 missense and 1 near-splice variants (Supplementary Table 1), but none of the missense variants seemed to have a high predicted impact on the protein function, all were known in gnomAD with frequencies ranging from 1.27e-4 to 1.06e-4 and their pathogenicity prediction scores were low. The near splice variant (NM_001184.3:c.6221+3G>A) was not predicted to alter splicing by HSF and SPiP.

Two additional possibly damaging variants of *ATR* were identified by cancer gene panel sequencing as part of their diagnostic work-up, both in female patients. Patient PED9545.1 had a variant of the initiation codon (NM_001184.3:c.1A>G - p.Met1?). Only two variants of this amino acid are reported in gnomAD in more than 233000 non cancer alleles. PED9545.1 developed BC at age 46 years. Her mother and her sister also developed BC at age 60yrs and 47yrs, respectively. Additionally, patient PED7847.1 had a near splice variant (NM_001184.3:c.2078+1G>A) altering the WT donor site of exon 9 of *ATR* that was predicted as “most probably affecting splicing” by HSF and SPiP gives it 98.41% of probability of affecting splicing. It is reported only once in gnomAD amongst 236364 non cancer alleles. PED7847.1 developed BC at age 57. No other incidences of BC are known in her family. Unfortunately, further segregation studies were not feasible in either of these families. The 4 potentially damaging variants identified in *ATR* are summarized in Table 1. The four patients in whom a potentially damaging *ATR* variant was identified are of Caucasian origin.

**Table 1:** Summary of possibly damaging variants of *ATR*

Patient	Sex	Histology	Family history	Sequencing method	Variant hg19 cDNA (NM_001184.3) Protein	gnomAD V2.1.1 non-cancer frequency
**PED2361.1**	M	Grade 3 invasive ductal carcinoma	Sporadic	Exome sequencing	chr3:g.142217574A>C c.5423T>G p.Leu1808*	Absent
**PED3315.1**	M	Grade 3 invasive ductal carcinoma	Sporadic	TS* in replication cohort	chr3:g.142168446T>G c.7762-2A>C	Absent
**PED9545.1**	F	*In situ* ductal carcinoma	Mother: BC (60) Sister: BC (47)	Cancer gene panel sequencing	chr3:g.142297546T>C c.1A>G p.Met1?	Absent
**PED7847.1**	F	Triple negative carcinoma	Sporadic	Cancer gene panel sequencing	chr3:g.142275224C>T c.2078+1G>A	4.23e-6 (1/236364)

Abbreviation: TS: Targeted sequencing.

### Impact of splicing variants

**Figure 1:**
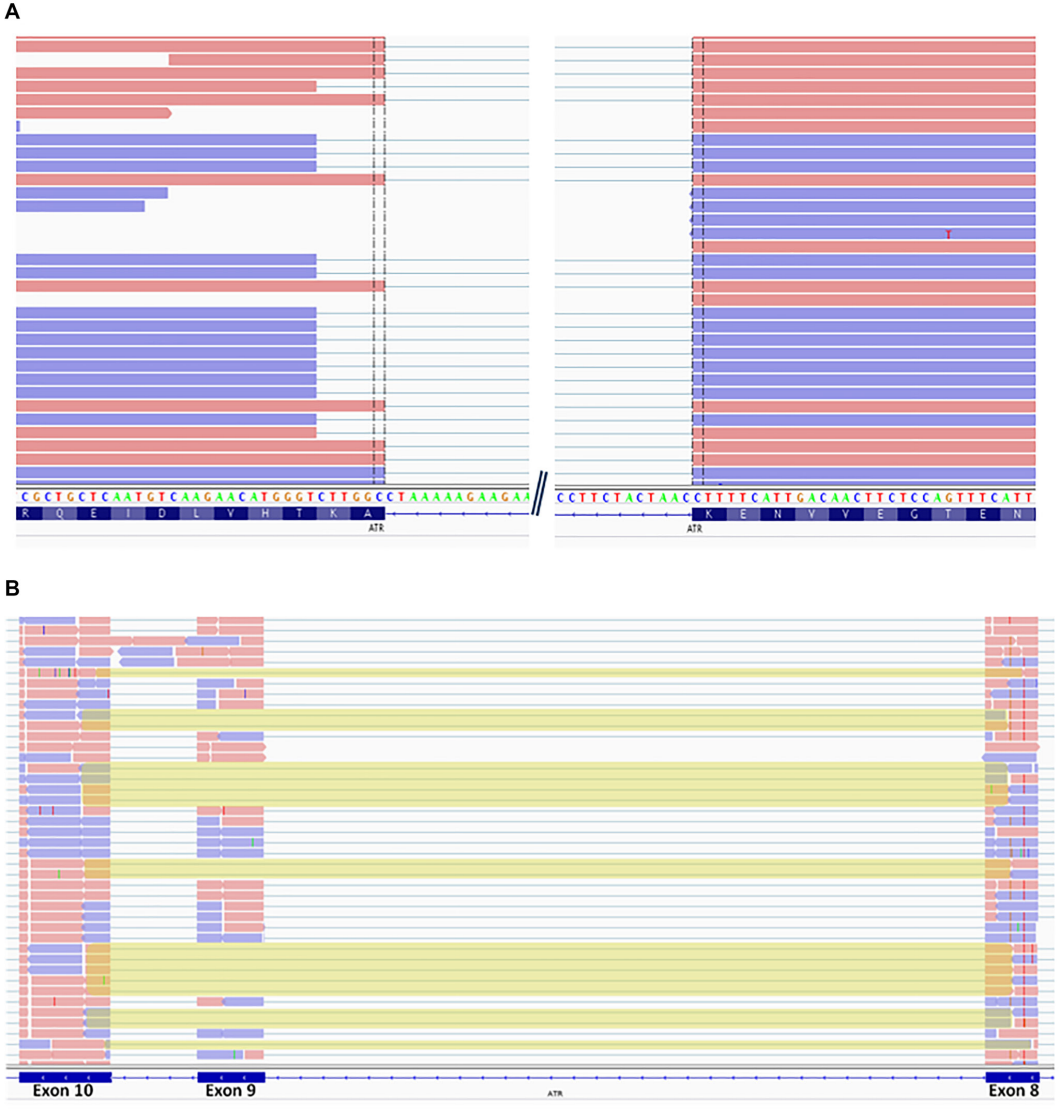
Sequencing of ATR cDNA. (**A**) IGV visualization of a subset of reads encompassing the last two exons of ATR cDNA for PED3315.1 carrying c.7762-2A>C variant. (**B**) IGV visualization of a subset of reads encompassing exons 8 to 10 of ATR cDNA for PED7847.1 carrying c.2078+1G>A variant.

In order to assess the impact of the two splicing variants identified in PED3315.1 and PED7847.1 (NM_001184.3:c.7762-2A>C and NM_001184.3:c.2078+1G>A, respectively), we sequenced the regions encompassing exons surrounding the variants on the cDNAs. Sequencing of the junction between the two last exons on PED3315.1 cDNA revealed a deletion of the first 6bps of the last exon in about 30 % of the transcripts (Figure 1A). Sequencing of an amplicon covering exon 7 to 13 of *ATR* in PED7847.1 cDNA showed the loss of exon 9 in about 25% of the transcripts (Figure 1B). These two alternative transcripts are absent from GTEX and Ensembl databases.

### Loss of heterozygosity (LOH)

LOH was assessed in PED2361.1 (MBC: pLeu1808*) by array Comparative Genomic Hybridization (CGH) (see Methods). None of the CGH probes show evidence of deletion at *ATR* locus in the tumor DNA of PED2361.1. We did not obtain tumor DNA from other patients.

### Impact of variants on ATR expression and kinase activity

**Figure 2:**
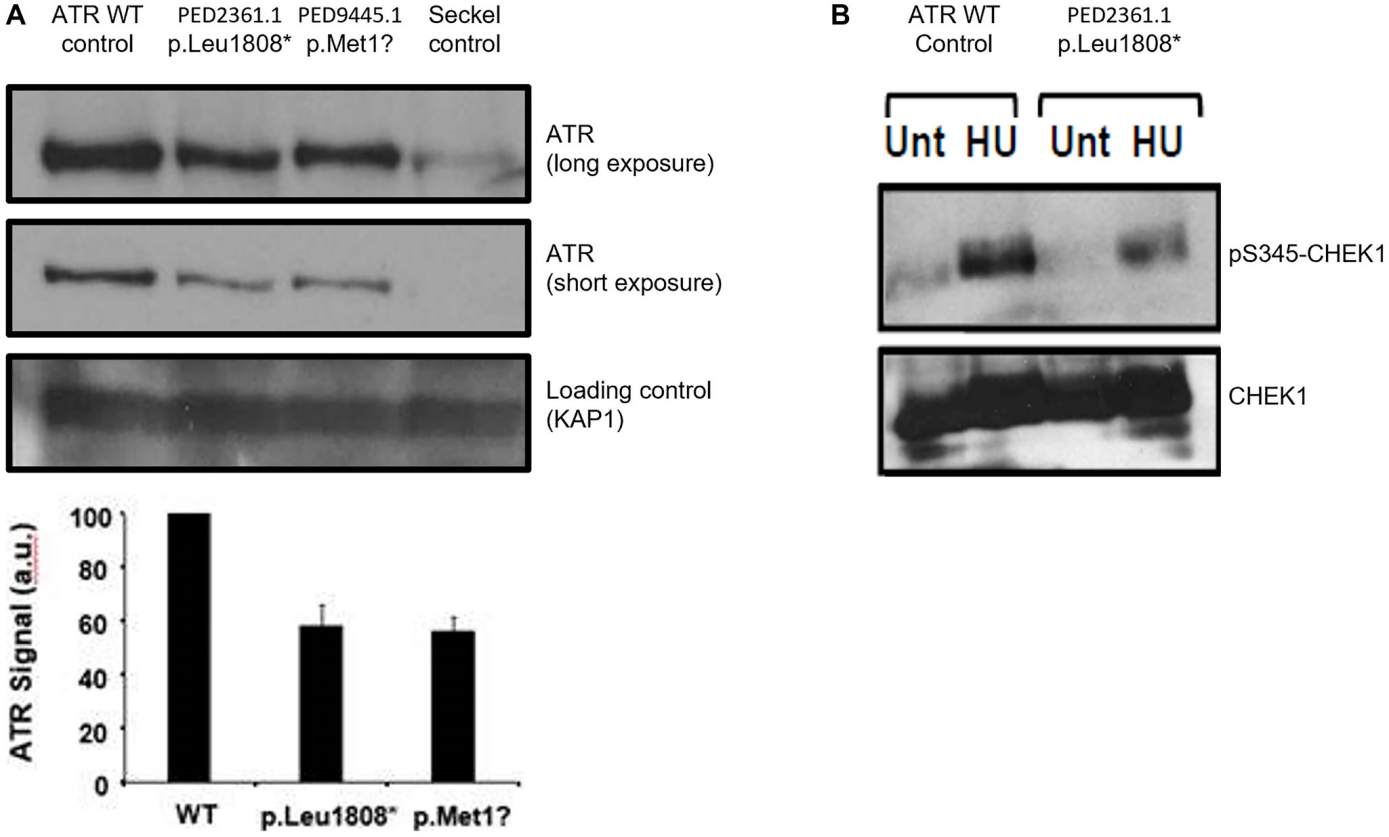
Impact of mutations on ATR expression and intra S phase checkpoint. (**A**) Western blotting of ATR expression in LCLs from patient PED2361.1 (p.Leu1808*) and patient PED9445.1 (p.Met1?). The Seckel control LCLs are from the ATR-Seckel patient described by Ogi T el al60 (p.M1159I and p.V2300Gfs*75). (**B**) Impact of variant of PED2361.1 on CHEK1 phosphorylation (Unt: without hydroxyurea treatment, HU: with hydroxyurea treatment).

Western blotting from lymphoblastoid cell lines (LCLs) derived from PED2361.1 (MBC: p.Leu1808*) and PED9545.1 (FBC: p.Met1?) showed a reduction of ATR expression in each of about 50 % compared with wild type (WT) controls (Figure 2A). No evidence of expression of a truncated ATR was found in LCLs from PED2361.1 (MBC: p.Leu1808*). Twice, the transformation to LCL failed for PED3315.1 (with the *ATR* splicing impacting variants).

CHEK1 is one of the main effectors of the S-G2/M cell cycle checkpoint following ATR activation during S-phase via direct phosphorylation by ATR on Serine 317 and 345. We assessed CHEK1 phosphorylation status following ATR activation by treatment with hydroxyurea (HU) in LCLs from PED2361.1 (MBC: p.Leu1808*). Western blotting showed an approximately 50% decrease in the phosphorylation of CHEK1 (pS345-CHEK1) following HU in the patient’s LCLs compared to control cells, indicating reduced ATR kinase function in these cells under these conditions (Figure 2B).

## DISCUSSION

ES and targeted NGS of constitutive DNA allowed us to identify 2 potentially damaging variants of *ATR* in 2 MBC cases out of 164 subjects with suspicion of cancer predisposition. Two additional variants were identified in FBC cases by cancer gene panel sequencing performed in a diagnostic procedure. These variants were either absent or very rare in control databases.

ATR plays a central role in the response to RS, DNA damage response (DDR) and cell cycle control making it a logical and likely candidate for cancer predisposition. Owing to its functional overlap with ATM, a DDR kinase already implicated in BC susceptibility, *ATR* has been previously tested as a potential candidate gene in two breast and ovarian cancer cohorts of respectively 126 and 54 patients without alterations of *BRCA1*/*2*. No deleterious *ATR* variants were identified in these studies [63, 64]. In 2016, an Australian team published the results of the sequencing of a cancer gene panel comprising *ATR* for 2000 BC cases with a strong familial history, WT for *BRCA1/2* and 1997 healthy controls. They identified 3 loss-of-function (LOF) variants in the cases and one in the control population [65]. Finally, a study close to our design identified a LOF *ATR* variant by ES in a BC patient who had been negatively tested for sequencing of a standard BC predisposition gene panel. Four additional LOF variants were found in a replication cohort of 2544 cases and 3 in 7652 controls. The comparison of frequencies was significant (*P* = 0.049) [66]. The authors concluded that *ATR* could be considered as a new candidate BC susceptibility gene. It expands the proportion of inherited BC that may be associated with rare variants in DDR pathways and supports the rare alleles model for susceptibility to cancer. This conclusion is nuanced by a more recent case-control study focusing on DDR genes in French population [67] in which 3 LOF variants out of 1207 female familial BC cases, and 4 LOF variants out of 1199 healthy controls were identified (*p* = 0,7). Overall, considering the last three cited studies and our work described here, 11 potentially damaging *ATR* variants have now been identified out of 5915 patients selected for BC predisposition.

**Figure 3:**
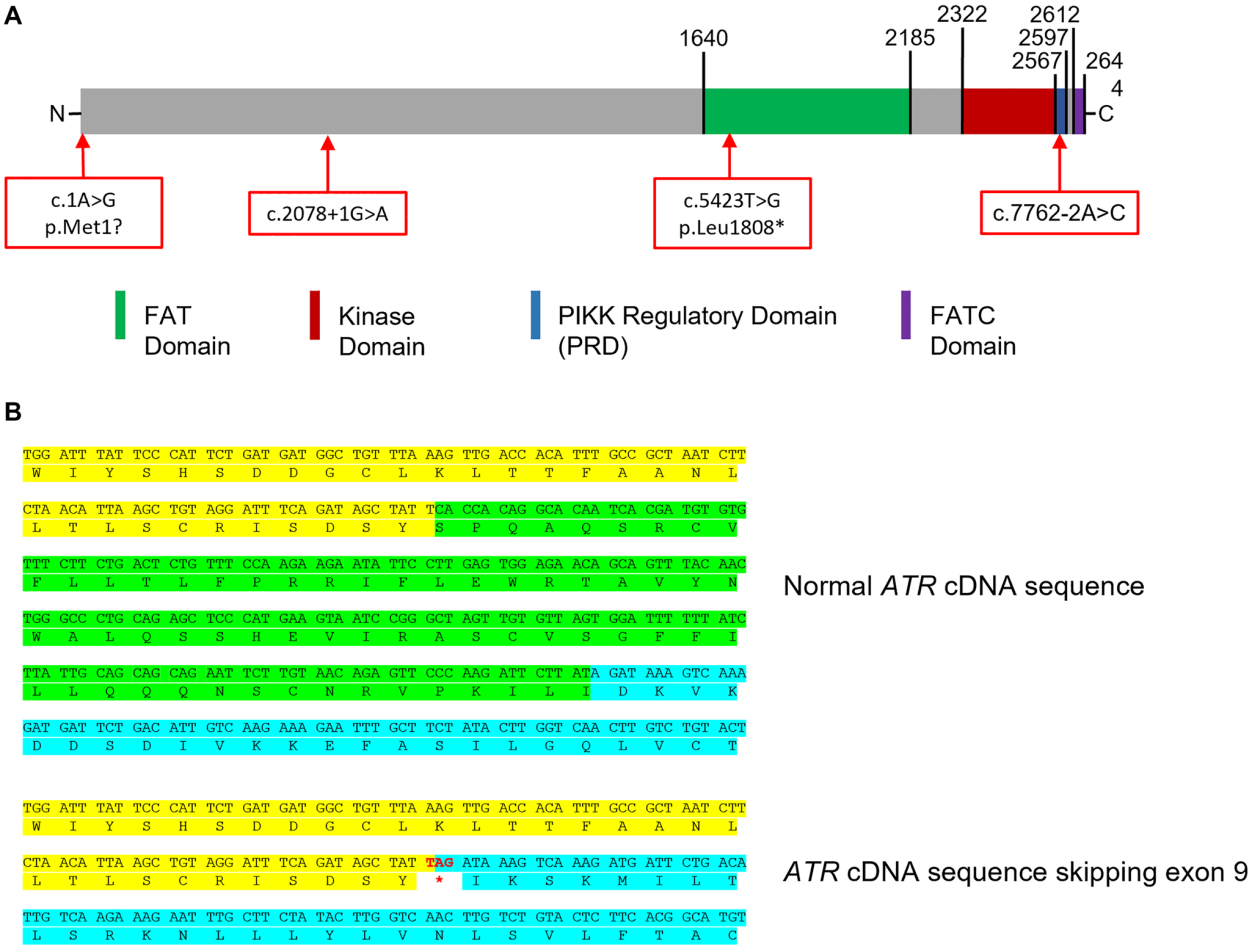
Impact of splicing variants. (**A**) Layout of C-terminal extremity of ATR. (**B**) Skipping of exon 9 creates a premature stop codon (Yellow: end of exon 8, green: exon 9, blue: beginning of exon 10).

None of the previous studies had evaluated the functional impact of *ATR* variants. In our study, we further explored the impact of some of the identified variants on *ATR* expression and ATR function by molecular and cellular experiments. First, we assessed the impact of splicing variants. The c.7762-2A>C variant identified in PED3315.1 was located in the consensus splicing acceptor site, just before the last exon of *ATR.* Generally, events affecting the last exon do not involve the nonsense-mediated mRNA decay or consequent loss of the protein, but typically to the presence of a truncated protein [68]. The consequences on protein activity could be minimal. However, the last *ATR* exon contains parts of the PRD domain (PIKK Regulation domain) and the FATC domain (Focal Adhesion Targeting, C-terminal domain), which are needful for the basal activity of ATR or its optimal activation in response to replication fork stalling. (Figure 3A). Loss of these domains is detrimental to the function of ATR [69, 70]. We showed that the c.7762-2A>C variant caused the loss of the first 6 bps of the last exon in about 30 % of the reads (Figure 1B). This deletion was in-frame and only led to the loss of two amino acids (p.Arg2588_Lys2589del), consequently this analysis did not allow us to conclude on the deleterious nature of this variant. Cell experiments could have helped to assess its impact but we were unable to obtain a cell line transformation from two samples of this patient.

The c.2078+1G>A variant identified in PED7847.1 was located in the consensus splicing donor site of exon 9 of *ATR.* Sequencing of an amplicon covering exon 9 to 13 of *ATR* cDNA showed the loss of exon 9 in about 25% of the reads (Figure 1B) and junction of exon 8 and 10 created a premature stop codon at position 693 (p.Ser629*) (Figure 3B), testifying of the high impact of this splicing variant.

We were able to generate LCLs from patients PED2361.1 (MBC: p.Leu1808*) and PED9545.1 (FBC: p.Met1?). Western blotting allowed us to demonstrate that ATR expression was reduced in LCLs from both patients, and that ATR-dependent phosphorylation of CHEK1 was impaired in PED2361.1 (MBC: p.Leu1808*) LCLs following treatment with HU. Our evidence suggests that these variant negatively impact ATR expression and in the instance of p.Leu1808* negatively impacts ATR kinase function.

When a tumor suppressor gene is implicated in a cancer predisposition, tumors often show loss of the WT allele, exposing the non-functional copy of the gene. In our case, array-CGH of constitutive versus tumoral DNA of PED2361.1 (MBC: p.Leu1808*) did not reveal a loss of heterozygosity (LOH) at *ATR* locus in the tumor. This data is consistent with the literature suggesting that *ATR* is essential for cell survival. Viable *ATR* variants are thought to be hypomorphic. Complete bi-allelic *ATR* inactivation causes excessive genomic instability leading to somatic and germ cell death and *Atr* −/− mouse models are not viable [43, 71, 72]. On the other hand, several studies have shown that *ATR* haploinsufficiency is responsible for alteration of DDR and cell cycle control in response to RS [73–77]. Furthermore, an increase in the incidence of tumors has been observed in *Atr* +/− mice in comparison with their non-mutated congeners [43]. Together, these data suggest that *ATR* haploinsufficiency could be involved in cancerous transformation.

Interestingly, a germline heterozygous missense (c.6431A>G; p.Gln2144Arg) variant of *ATR* has been identified in several members of a family with a syndrome associating skin telangiectasias and anomalies of hair, eyebrows, tooth and nails. Ten of the 24 affected individuals developed an oropharyngeal cancer from the age of 30 for the earliest one. This variant did not result in a reduction of *ATR* expression at mRNA or protein level, but sequencing of the tumor of one of the affected individuals showed a loss of the wild-type *ATR* allele [78].

Considering the essential function of ATR in controlling genome integrity, it could be important to enhance cancer surveillance in ATR Seckel patients (carrier of constitutional homozygous or compound-heterozygous hypomorphic variants of *ATR*). Their parents, heterozygous carriers of pathogenic *ATR* variants, should also be monitored for cancer predisposition as it is done for heterozygous carriers of *ATM* pathogenic variant that cause ataxia-telangiectasia in a homozygous state [79–81]. It may be surprising that no malignant lesions have been reported to-date in Seckel syndrome patients with *ATR* variants nor in a humanized mouse model of *Atr*-Seckel syndrome [59, 60]. This might be related to the large magnitude of genomic instability mediated via bi-allelic disruption of *ATR* in those specific contexts. Additional genomic stress due to the alteration of other tumor suppressor genes or activation of oncogenes during early tumorigenesis could lead to cell death rather than transformation. This theory is further supported by studies showing that *Atr* loss is synthetic lethal with disruption of other tumor suppressor genes like *Tp53* [82] or oncogene-driven replication damage [47–50] and ongoing clinical trials on *ATR* inhibitors. Thereby, cancer predisposition associated with ATR deficiency should follow the model of obligate haploinsufficiency described by Berger et al. [83].

Our results and the data from the literature support a model in which moderate RS due to the lack of one allele of *ATR* enables cells to survive, but likely generates a latent genetic instability that may initiate and/or drive cancerous transformation. The specifics of this transformation mechanism seems to diverge from that described by Tanaka and collaborators in the family with predisposition to oropharyngeal cancer since the identified missense variant did not cause a decrease in the expression of ATR and was associated with LOH in the tumor [58].

In conclusion, this work highlights the possible implication of *ATR* variants in male and female BC predisposition and shows the importance of extended genetic analysis in unsolved extreme phenotype cancer cases to identify rare alleles of biologically relevant candidate genes of cancer predisposition. However, as shown by cases-controls studies which have identified LOF *ATR* variants in control patients, [66, 67], an incomplete penetrance could be evoked. This is well known with other cancer predisposition genes and large-scale studies are needed to evaluate the penetrance of cancer susceptibility genes in general population [84]. More functional studies could also help to determine if *ATR* variants play a major role in cancer initiation, whether they are part of a cluster of molecular events each having a weak effect, or whether they are only a modifying element that accelerates the process of tumorigenesis in coordination with additional somatic events.

## MATERIALS AND METHODS

### Patients

The index patient originated from the EX^2^TRICAN project, proposing ES in extreme cancer phenotypes using different strategies: (i) trio ES for early onset sporadic cases or sporadic cases with multiple primary malignancies for comparative index case-parents ES strategy, originally developed to detect *de novo* variants; (ii) ES of two distant cases in families with a strong aggregation of cancer cases; (iii) solo ES with familial segregation of candidate variants for very rare cancer types or if only the proband is available for ES. Among patients included in this study, there were 3 patients from families with multiple cases of MBC.

In order to give emphasis of the possible role of *ATR* in the predisposition to BC and in particular MBC, we constituted a replication cohort of 86 MBC cases, 28 MBC related women and 47 women with very high risk of BC according to the BOADICEA software [52]. Patients with MBC have been recruited from Dijon University Hospital, the anti-cancer Center Georges François Leclerc (CGFL Dijon) and a national collaboration call, and the MBC related women and women with very high risk of BC families have been recruited from Dijon University Hospital and the CGFL Dijon. All patients of this cohort have been tested negatively at least for the more penetrant BC predisposition genes (*BRCA1, BRCA2* and *PALB2*).

Finally, after the discovery of the first *ATR* variant by ES, *ATR* has been included in the cancer gene-panel used for genetic predisposition to cancer at CGFL Dijon. This lead to the identification of two *ATR* variants in patients PED9545.1 and PED7847.1. They were negative for the following predisposition genes : *BRCA1, BRCA2, PALB2, PTEN, TP53, RAD51C, RAD51D, CDH1, EPCAM, MLH1, MSH2, MSH6, PMS2, APC, MUTYH, AXIN2, GREM1, NTHL1, POLD1, POLE, SMAD4, STK11, RET, MEN1, SDHAF2, SDHB, SDHC, SDHD, VHL, FLCN, WT1, BAP1, CDKN2A, CDK4, PRSS1, NF1, PTCH1, PTCH2*.

### DNA extraction

From whole blood, genomic DNA (gDNAs) was extracted from 3–5 mL of whole blood using the Gentra Puregene kit (Qiagen GmbH, Hilden, Germany) following the protocol recommended by the supplier.

From fresh frozen tissue, twenty milligrams of frozen tissue were digested overnight at 56°C with moderate agitation in 180 μL of ATL buffer and 20 μL of proteinase K from the QIAamp DNA Mini kit (Qiagen GmbH, Hilden, Germany). The genomic DNA is then extracted from this lysate according to the DNA Purification from Tissues protocol.

### RNA extraction and cDNA synthesis

Total RNA was extracted from whole blood collected in a PAXgene tube (Preanalytics GmbH, Hombrechtikon, Switzerland) using the PAXgene Blood RNA kit (Preanalytics GmbH, Hombrechtikon, Switzerland) following the standard protocol. cDNA was obtained using the QuantiTect Reverse Transcription kit (Qiagen GmbH, Hilden, Germany).

### Exome sequencing (ES)

Three micrograms of genomic DNA were subjected to exome capture using the SureSelect Human All Exon V5 kit (Agilent Technologies, Santa Clara, CA, USA). The resulting library was sequenced on a HiSeq 2000 (Illumina, San Diego, CA, USA) as paired-end 101 bp reads. FASTQ files were aligned to a human genome reference sequence (GRCh37/hg19) using BWA (Burrows-Wheeler Aligner; V.0.7.15). All aligned read data were subject to the following steps: (1) duplicate paired-end reads were removed by Picard 2.4.1, (2) base quality score recalibration was performed with Genome Analysis Toolkit (GATK; V.3.7). Haplotype Caller from GATK was used to perform the variant calling. Variants with a quality score >30 and alignment quality score >20 were annotated with the SNPEff tool. Variants present at a frequency above 1% in dbSNP 150 and in the gnomAD database or present from 100 exomes of unaffected individuals were excluded. Remaining variants, supported by ≥3 reads and ≥10% of total reads, were reviewed by focusing on protein-altering and splice-site DNA changes absent in Exome Variant Server and gnomAD. In fist line, interpretation was limited to variants affecting genes known to be responsible for syndromic or non-syndromic cancer predisposition in the Online Mendelian Inheritance in Man database (OMIM). Then analysis was focused on a list of 661 cancer predisposition genes, DNA repair genes or drivers genes in cancer development drawn from the COSMIC Census database, published cancer predisposition and DNA repair genes lists, and literature review. Priority has been given to LOF variants (out of phase insertions or deletions and non-sens substitutions). *In silico* prediction tools have been used to assess the pathogenicity of splicing variants (HSF and SPiP) [85, 86] and missense variants : Combined Annotation Dependent Depletion (CADD) [87], Polymorphism Phenotyping v2 (PolyPhen2) [88], Genomic Evolutionary Rate Profiling (GERP) [89]. These prediction tools have been chosen for their complementarity as they involve different analysis methods. Sequencing data of each patient were analyzed individually, no stringent threshold was defined on the basis of scores given by these prediction. Prediction tools were used as an aid for prioritization and interpretation of missense variants, but were not used to filter out missense variants.

### Targeted NGS

All coding exons of the *ATR* gene have been amplified with 17 primers pairs. Primers have been designed thanks to the application ExonPrimer (https://ihg.helmholtz-muenchen.de/ihg/ExonPrimer.html) with a maximum amplicon length of 9 kb. They are listed in Supplementary Table 2. Primer pairs for cDNA sequencing were designed manually thanks to the Primer 3 software (https://probes.pw.usda.gov/cgi-bin/batchprimer3/batchprimer3.cgi) in order to amplify the region encompassing exons surrounding the variants. After checking of PCR products by agarose gel electrophoresis, all amplicons of the same patient were pooled in a single tube and purified with AMpure XP magnetic beads (Beckman Coulter Inc., Brea, CA, USA). Sequencing libraries were prepared with the Nextera XT kit (Illumina, San Diego, CA, USA) and sequenced on Miseq in 150 bases length paired end reads. Raw data analysis is performed with the same bioinformatics program as for exome data analysis with some adaptations for targeted sequencing. For cDNA sequencing, raw data have been aligned with STAR software (v2.5.2b) and IGV (Integrated Genome Viewer, v2.5.2) has been used to visualize the reads and generate graphical representations.

### Sanger sequencing

For Sanger sequencing, PCR were done using the HotStar Taq DNA polymerase kit (Qiagen GmbH, Hilden, Germany). PCR products have been controlled on agarose gel and then purified with the ExoSAP-IT kit (Thermo Fisher Scientific Inc, Waltham, MA, USA). Sequence reactions were prepared and purified using BigDye Terminator v1.1 and BigDye XTerminator kits (Thermo Fisher Scientific Inc, Waltham, MA, USA) following standard protocol. The sequencing products were then analyzed by capillary electrophoresis on an ABI PRISM 3130 × l genetic analyzer (Thermo Fisher Scientific Inc, Waltham, MA, USA). Results were analyzed using the FinchTV software (Digital World Biology LLC, Seattle WA, USA).

### Search for loss of heterozygosity by array-CGH

Germline heterozygous variants of tumor suppressor genes are often followed by the loss of the remaining WT allele in the tumor cells. This loss of heterozygosity (LOH) was assessed in a fresh frozen tumor sample of PED2361.1 by array Comparative Genomic Hybridization (array-CGH). Beforehand, the amount of cancer cells in the samples has been evaluated at 80% by a pathologist. Comparative array-CGH between constitutional and tumor DNA has been performed on a SurePrint G3 Human 4 × 180 k chip (Agilent Technologies, Santa Clara, CA, USA) containing 12 probes covering *ATR*, according to the supplier’s recommendations. The genomic DNAs were digested with AluI/RsaI restriction enzymes for 2 hours at 37°C. Then DNAs were marked respectively Cyanine5 or Cyanine3 by random priming using the DNA labeling Plus kit (Agilent Technologies, Santa Clara, CA, USA), and then purified on QIAquick PCR Purification columns (Qiagen GmbH, Hilden, Germany). Effectiveness of the labeling was verified with Nanodrop quantification (Thermo Fisher Scientific Inc, Waltham, MA, USA). The labeled DNAs are then co-hybridized on the slide for 40 hours at 65°C. with stirring by constant rotation. After washing, the slide was read by the G2565BA scanner (Agilent Technologies, Santa Clara, CA, USA). Feature Extraction and Genomic WorkBench 6.5 software (Agilent Technologies, Santa Clara, CA, USA) were used to collect and analyze the data.

### LCL culture

LCL were established from a heparinized blood sample by the European Collection of Authenticated Cell Cultures laboratory and then transferred to the Human DNA Damage Response Disorders (HDDRD) laboratory of Sussex University where they were maintained in RPMI 1640 medium added of 15% fetal bovine serum, antibiotics and L-glutamine. For the analysis of CHEK1 phosphorylation after induction of genotoxic stress, cells were treated for 2 hours with 500 μM of hydroxyurea before the extraction proteins extraction.

### Western blot

For protein extraction, cells were incubated 15 minutes on ice in RIPA buffer (Cell Signaling Technology Inc., Danvers, MA, USA) added of protease inhibitor cocktail (Merck KGaA, Darmstadt, Germany) and phenylmethylsulphonyl fluoride (Merck KGaA, Darmstadt, Germany) and then centrifuged 15 minutes at 4°C. Protein assay was performed using a Bovine Serum Albumin Standard Series and the BCA Protein Assay Kit (Thermo Fisher Scientific Inc, Waltham, MA, USA). The absorbance reading (562 nm) was performed on a Multiskan Go 96-well plate reader spectrophotometer (Thermo Fisher Scientific Inc, Waltham, MA, USA). An equal amount of protein of each sample were separated by electrophoresis in acrylamide gel and then transferred on polyvinylidene difluoride membranes (Merck KGaA, Darmstadt, Germany). Membranes were incubated overnight at 4°C with primary antibodies (ATR, Santa Cruz Biotechnology, Inc., Dallas, TE, USA; CHK1 and P-CHK1 (Ser345), Cell Signaling Technology Inc., Danvers, MA, USA). Then membranes were washed and incubated with secondary antibody coupled with peroxidase (Cell Signaling Technology Inc., Danvers, MA, USA). Revelation of blots was performed with the Clarity Western ECL Substrate (Bio-Rad Laboratories Inc., Hercules, CA, USA) and images were realized with the ChemiDoc MP instrument (Bio-Rad Laboratories Inc., Hercules, CA, USA).

## SUPPLEMENTARY MATERIALS



## Abbreviations

MBC: Male Breast Cancer
FBC: Female Breast Cancer
ES: Exome Sequencing
TS: Targeted Sequencing
FFPE: Formalin Fixed and Paraffin Embedded
LCL: Lymphoblastoid Cell Line
FPC: Fibroblast Primary Culture
CGH: Comparative Genomic Hybridization
LOH: Loss of Heterozygosity
NGS: Next Generation Sequencing
RS: Replication stress
WT: Wild Type
DDR: DNA Damage Response
LOF: Loss-of-function
