# Supplementary material for: The “extreme phenotype approach” applied to male breast cancer allows the identification of rare variants of ATR as potential breast cancer susceptibility alleles
Source: Oncotarget. 2023 Feb 7;14:111–25. doi: 10.18632/oncotarget.28358 (PMC9904323; doi:10.18632/oncotarget.28358)
Supplement: Supplementary file 1 [file oncotarget-14-28358-s001.pdf]

The "extreme phenotype approach" applied to male breast cancer allows the identification of rare variants of ATR as potential breast cancer susceptibility alleles

SUPPLEMENTARY MATERIALS

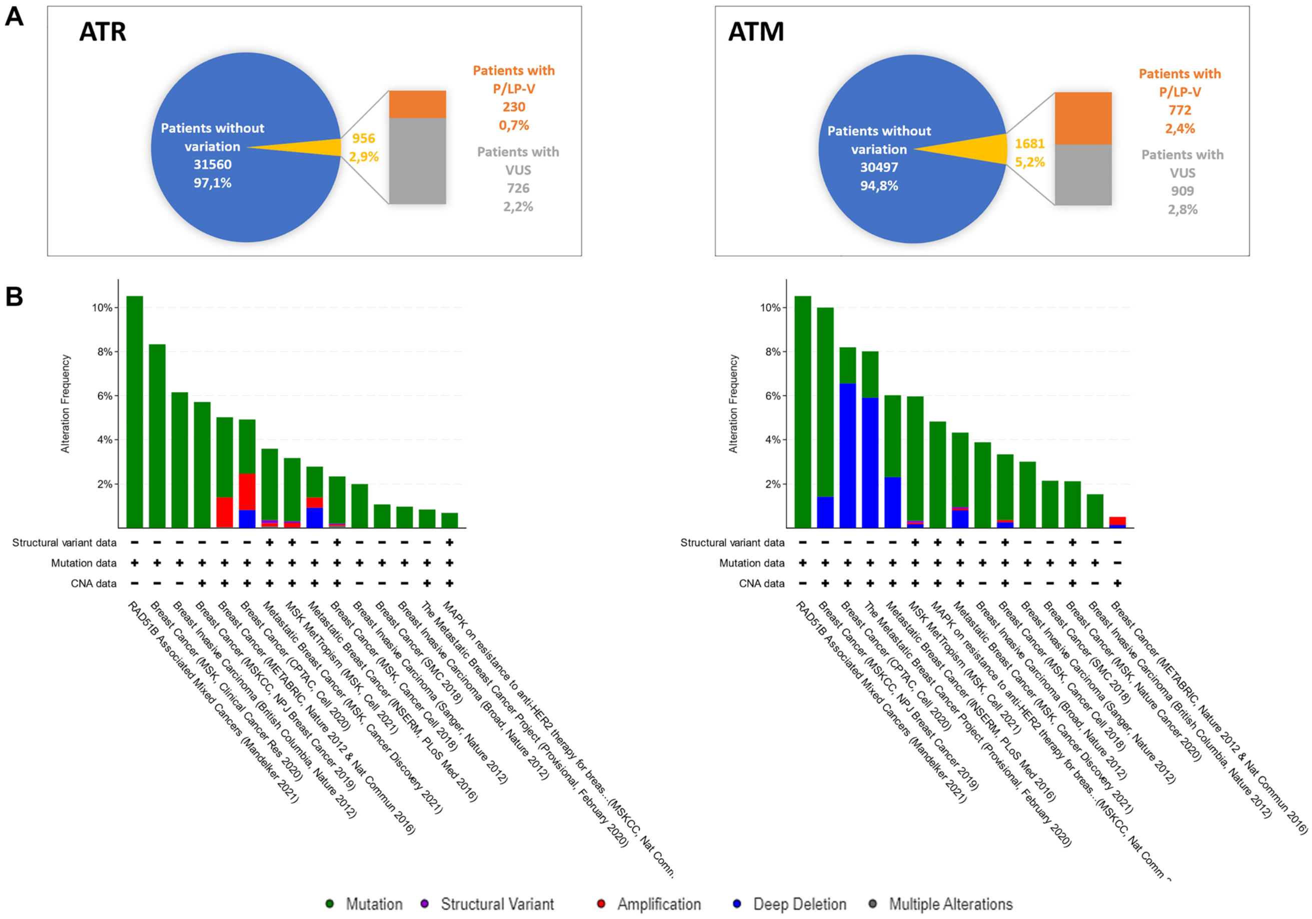

Supplementary Figure 1: Comparison of somatic variation rates of *ATR* and *ATM* in BC studies from cBioPortal. (**A**) Proportion of patients with somatic variation of *ATR* and *ATM* in BC studies (P/LP-V: Pathogenic or likely pathogenic variations). (**B**) Frequency and type of variation identified in BC studies.

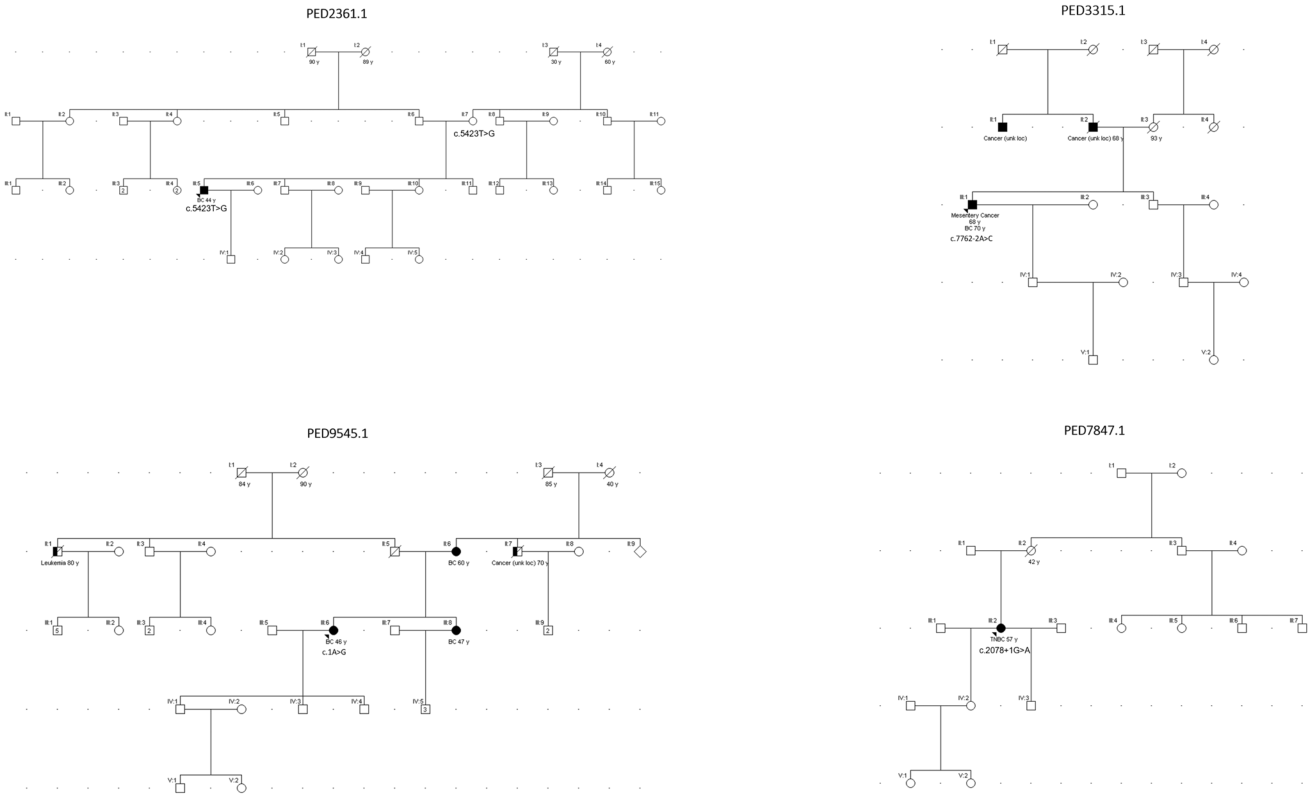

Supplementary Figure 2: Genealogical trees. Abbreviations: BC: breast cancer; TNBC: triple negative breast cancer; unk loc: unknown localization; y: years.

Supplementary Table 1: Other variants identified in the replication cohort

Genomic variant	cDNA Variant (NM_001184.3)	Protein variant	gnomAD frequency	CADD	PolyPhen2	GERP	Grantham score	HSF prediction
chr3:g.142203979C>T	c.6221+3G>A	.	3.13e-4 (84/268202)	8.693	–	0.322	–	No significant splicing motif alteration Probably no impact on splicing
chr3:g.142261533T>C	c.3424A>G	p.Ser1142Gly	5.53e-4 (148/267744)	14.790	0.044	5.470	56	–
chr3:g.142272098A>G	c.2776T>C	p.Phe926Leu	1.06e-3 (284/268164)	20.300	0.355	5.350	22	–
chr3:g.142232392T>C	c.4592A>G	p.His1531Arg	1.27e-4 (34/168182)	22.200	1	4.890	29	–
chr3:g.142272170A>G	c.2704T>C	p.Ser902Pro	6.08e-4 (163/268262)	12.880	0.006	5.530	74	–
chr3:g.142178115T>C	c.7303A>G	p.Ile2435Val	2.5e-4 (67/268186)	0.200	0.000	2.680	29	–

Abbreviation: HSF: Human splicing finder.

Supplementary Table 2: List of PCR primers

Primers	Primers sequence	Amplicon size (bp)	% GC	Amplicon location (hg19)
**Targeted next generation sequencing of *ATR***				
ATR_ex1_F	CGGGTCCTATGCAGAAAAGAC	410	66	chr3:142297311-142297720
ATR_ex1_R	TATCAACGGGCACTCTGAGG			
ATR_ex2-5_F	GAGAAGTTCAGGTCTAGGCTTTG	7276	36	chr3:142279958-142287233
ATR_ex2-5_R	CCCTTGGCTACATTTAGAAAGATG			
ATR_ex6-12_F	GTGGCTAAGTTGCCATATTTCAG	7660	37	chr3:142272114-142279773
ATR_ex6-12_R	AACACTTTTAGCTGCAACCAGAG			
ATR_ex13-16_F	GAAGGTATACTTGGCAGCATTTG	6196	37	chr3:142266290-142272485
ATR_ex13-16_R_2	TCAATGCTAATATATGTTGTTAATGCC			
ATR_ex17-20_F	CATGTGAAGATATATGCTTTTGGAG	6985	33	chr3:142254782-142261766
ATR_ex17-20_R	AAAAGTCATGGATTCATGGTAGC			
ATR_ex21_F	GCTGTGGGATGATTCTAGTTTTG	1313	32	chr3:142253305-142254617
ATR_ex21_R	AAATTCATGAGGGTCTTAGGCTC			
ATR_ex22-24_F	AATCTTCACTGGCTTGAGAATTG	5710	36	chr3:142238014-142243723
ATR_ex22-24_R	AGAATTTAACCCAATGAGCAGAC			
ATR_ex25-27_F	AGCTAGACACTGAAGTCACCCAG	4153	37	chr3:142230625-142234777
ATR_ex25-27_R	TTCTCAGTGAAAAGGCCACAC			
ATR_ex28-30_F	AGTTGGAGCAAGACTCTCTGAAC	5115	37	chr3:142222013-142227127
ATR_ex28-30_R	GACAGAAGCTGTTAAGTGGTAGACTG			
ATR_ex31-34_F	GAGTTATTGGAATTGCTTACCTCTG	4005	36	chr3:142214958-142218962
ATR_ex31-34_R	CCTTGAAATGGGAAGGTATCTG			
ATR_ex35_F	AACCTGTGGCTACAGAGAGCC	639	31	chr3:142211743-142212381
ATR_ex35_R	ATTACTGGGATGAAGGGTAGTGG			
ATR_ex36_F	GCTATTCCTTTTCCACATCAAAG	571	36	chr3:142203711-142204281
ATR_ex36_R	ATGATTGCTGTGTTGGTGTGAG			
ATR_ex36_F2	TTTCCAAAACACATGTCTATATCAC	498	36	chr3:142203761-142204258
ATR_ex36_R2	ATTGAGTCTGCTTTTGACTTTGC			

ATR_ex37-39_F	TTTCCCAGAAATAGAATGATTGG	2871	31	chr3:142186525-142189395
ATR_ex37-39_R	TCTCGGAAAATTCTTAATTCACAC			
ATR_ex40-41_F	ATGACAGAGGTTAAAATGGGATG	2032	32	chr3:142183493-142185524
ATR_ex40-41_R	GGTACCTCAAATGGTTAAATTAATGG			
ATR_ex42-45_F	AAAAGAAGGACCTACCCAGGAC	5035	33	chr3:142176126-142181160
ATR_ex42-45_R	TAGATAGCAGGGGTGGGAGG			
ATR_ex46-47_F	ATGGCACATAGAACCCATCTTAC	4335	40	chr3:142167997-142172331
ATR_ex46-47_R	GACTTGCTTGTTTCTTGCAAATATAG			
**Targeted next generation sequencing of *ATR cDNA***				
ATR_cDNA_ex46-47F	TACATGATCCTCTTGTGGAATGG	393	37	chr3:142168141-142172058
ATR_cDNA_ex46-47R	TCAGAGAGAAATAACAGTTGCTGAG			
ATR_cDNA_ex7-13F	GACCTGGAGGCAACCATTGA	970	37	chr3:142272233-142278153
ATR_cDNA_ex7-13R	TTGCGGCCCTTCCAATATCC			
